# Effect of Season on the Characteristics of Warmblood Stallion Spermatozoa Stored in a Liquid State at 5 °C

**DOI:** 10.3390/ani15071035

**Published:** 2025-04-03

**Authors:** Anna Dziekońska, Agata Szczepańska, Anna Wysokińska

**Affiliations:** 1 Department of Animal Biochemistry and Biotechnology, University of Warmia and Mazury in Olsztyn, Oczapowskiego 5, 10-719 Olsztyn, Poland; a.szczepanska@ibb.waw.pl; 2 Laboratory of Animal Molecular Physiology, Institute of Biochemistry and Biophysics of the Polish Academy of Sciences, Pawińskiego 5A, 02-106 Warsaw, Poland; 3 Faculty of Agricultural Sciences, Institute of Animal Science and Fisheries, University of Siedlce, Prusa 14, 08-110 Siedlce, Poland; anna.wysokinska@uph.edu.pl

**Keywords:** stallion sperm, season, liquid state, quality, mitochondria

## Abstract

The aim of this study was to investigate the effect of season (breeding vs. non-breeding) on the characteristics of warmblood stallion spermatozoa during liquid storage. Ejaculates were collected from eight stallions during the breeding and non-breeding seasons (March–July and September–December, respectively) and were diluted in an EquiPro extender. Semen was stored for up to 96 h at 5 °C. Analysis of stored sperm included the assessment of motility (CASA system), acrosomes with normal apical ridges (NAR), plasma membrane integrity (PMI), mitochondrial membrane potential (MMP), and adenosine triphosphate (ATP) content. In sperm stored for up to 24 h, the values of the following parameters were higher (*p* ≤ 0.05) in March-July than in September–December: progressive motility (PMOT), PMI, MMP, beat cross frequency (BCF), and linearity (LIN). The values of the studied parameters decreased during storage, and the observed changes were affected by season. The values of NAR, PMI and MMP, average path velocity (VAP), straight line velocity (VSL), and curvilinear velocity (VCL) decreased (*p* ≤ 0.05) after 24 h of storage during the breeding season, whereas a significant decrease in these parameters was observed after prolonged storage (48 h or longer) during the non-breeding seasons. Cooled sperm of warmblood stallions collected during the breeding season was characterized by higher initial quality than the sperm collected during the non-breeding season. However, sperm sampled during the non-breeding season appeared to be more resistant to cold shock, which increases their suitability for cold storage at 5 °C. Stallion sperm sampled in both seasons were characterized by similar suitability for liquid storage. Further research is needed to assess the fertilizing capacity of stored sperm.

## Simple Summary

Seasonal variations in sperm quality have been reported in many animal species. The impact of season on stallion sperm quality was demonstrated for fresh and cryopreserved semen. However, the effect of seasonality on stallion sperm stored in a liquid state remains insufficiently investigated. Previous studies have primarily focused on the assessment of motility and DNA integrity. Therefore, the aim of this study was to comprehensively assess the effect of season on the quality of warmblood stallion sperm stored in a liquid state for up to 96 h at 5 °C. Analyses of sperm quality included the assessment of motility, integrity of plasma and acrosomal membranes, mitochondrial efficiency, and adenosine triphosphate (ATP) content. It was found that spermatozoa collected during the breeding season (March–July) and stored for up to 24 h were characterized by higher quality than those collected outside the breeding season (September–December). Nevertheless, prolonged storage in this study did not induce significant differences in the quality of spermatozoa between the compared seasons. These findings may indicate that the spermatozoa of warmblood stallions collected outside the breeding season are more resistant to low temperature than those collected during the breeding season, which confirms the suitability of the former for cold storage.

## 1. Introduction

Cooled or cryopreserved semen is routinely used for artificial insemination (AI) in horses. For practical reasons, chilled semen is more recommended for equine AI than cryopreserved semen because it increases the chances of fertilization success [1,2,3]. Stallion semen is usually stored in a liquid state at a temperature of 4–5 °C for up to three or four days [4]. However, recent research has shown that ejaculated stallion spermatozoa maintain their fertilizing capacity for up to seven days when stored at a temperature of 17 °C [4].

The effectiveness of AI depends on semen quality, which is determined by many factors, including semen storage conditions, individual traits, breed, season, and the stallions’ age [5,6]. In animals, reproductive seasonality is usually associated with variations in photoperiod and ambient temperature, and these factors significantly affect semen quality in many animal species, including horses [7,8,9,10]. Horses are long-day breeders, and they reproduce in seasons with long periods of daylight, during which circulating testosterone levels and spermatogenesis increase in stallions [11,12]. Stallion semen can be used for reproductive purposes throughout the year, but its biochemical composition and content of selected nutrients fluctuate considerably across seasons [13,14]. Numerous studies conducted on fresh and cryopreserved semen have shown that seasonal changes in its biochemical composition can affect the quality and fertilizing capacity of ejaculates [13,15,16]. The effect of season on the quality of chilled semen has been less extensively researched [17,18]. The results of studies investigating the impact of seasonality on the quality of fresh and stored stallion semen are inconclusive, which may be attributed to differences in geographic location and climatic factors [15,19,20]. These discrepancies can also result from the use of different analytical methods in studies examining the quality of stallion semen [17,20,21].

Based on the results of the literature review, the authors hypothesized that season may have a significant influence on sperm quality in warmblood stallions in Poland and in other countries of Central Europe due to considerable seasonal differences in day length and temperature differences observed throughout the year. Therefore, the aim of this study was to determine the effect of season (breeding vs. non-breeding) on the characteristics of warmblood stallion sperm stored in a liquid state at 5 °C for different periods of time (0 h, 24 h, 48 h, 72 h, 96 h). The quality of stored spermatozoa was assessed based on changes in sperm motility (computer-assisted sperm analysis, CASA system), acrosome integrity, plasma membrane integrity (PMI), mitochondrial membrane potential (MMP), and adenosine triphosphate (ATP) content.

## 2. Materials and Methods

### 2.1. Animals and Ejaculate Collections

A total of eight reproductively sound, mature warmblood stallions (aged 10–21 years) were used for the experiment. The animals originated from two stallion studs in Poland. Four horses were provided by Stack Stallions in Łąck (Region of Mazowsze, Poland), and four were provided by Marek Romanowski’s stable in Wozławki (Region of Warmia and Mazury, Poland). The stallions had unlimited access to water and were fed in line with standard housing protocols.

Ejaculates were collected at regular intervals (once or twice per week) from each stallion during the breeding season (March–July, *n* = 16) and the non-breeding season (September–December, *n* = 18) using a Missouri-style equine artificial vagina (Missouri Model AV, Nasco, Ft. Atkinson, WI, USA), either on a dummy mare or on a mare in estrus, according to standard procedures. The following number of samples was obtained for analytical purposes: two ejaculates were collected from each stallion during the breeding season, whereas two ejaculates (per animal) from six stallions and three ejaculates (per animal) from two stallions were sampled during the non-breeding season.

During a preliminary assessment of sperm samples, sperm concentrations were determined using a Bürker chamber (Equimed-Medical Instruments, Cracow, Poland), and sperm motility was evaluated under a microscope [19].

### 2.2. Fresh Semen Processing Procedure

Immediately after collection, the gel fraction was removed from the ejaculate by filtering through sterile gauze. Each ejaculate was routinely assessed for quality parameters, including ejaculate volume, sperm concentration (Con), total sperm motility (TMOT), and total protein content of seminal plasma (SP). The volume of the gel-free ejaculate was assessed with a glass cylinder. Sperm concentration was determined using a Bürker counting chamber (Equimed-Medical Instruments, Kraków, Poland). To separate SP, each ejaculate portion was centrifuged at 10,000× *g* for 10 min (at room temperature). The total protein content of SP was measured according to the method proposed by Lowry et al. [22], with bovine serum albumin (BSA, IBSS BIOMED S.A., Kraków, Poland) as the standard. Total sperm motility (TMOT) in raw semen was assessed subjectively. For this purpose, semen samples were diluted with a commercial equine extender, EquiPro (Minitübe, Tiefenbach, Germany), to a concentration of 20 to 30 × 10^6^ spz/mL [23]. Then, aliquots (5 µL) of diluted semen samples were placed on prewarmed slides, covered with glass cover slides, and examined under a light microscope with an attached heated stage (37 °C) at 200× magnification.

Semen (sperm-rich fraction) with total motility above 70% was used for liquid storage. Semen was diluted with the EquiPro extender to 30 × 10^6^ sperm/mL and transported (approx. 3–4 h) at 17 °C (Thermobox, Minitüb Gmbh, Tiefenbach, Germany) to the laboratory of the Department of Animal Biochemistry and Biotechnology of University of Warmia and Mazury in Olsztyn, Poland. In the laboratory, diluted semen was placed in a refrigerator at 5 °C and stored at that temperature for up to 96 h.

### 2.3. Analysis of Sperm Parameters After Cooling

Cooled spermatozoa were analyzed after 2 h (time—0 h), 24 h, 48 h, 72 h, and 96 h of storage at 5 °C.

#### 2.3.1. Motility Evaluations

Sperm motility parameters were analyzed in the CASA system (manufactured by Hamilton Thorne, HTR, IVOS version 12.3; Beverley, MA, USA). A droplet (approx. 5 μL) of semen was placed in a Makler counting chamber (Sefi-Medical Instruments Ltd., Haifa, Israel) pre-warmed to 37 °C and analyzed in six fields of view per sample. A minimum of 200 spermatozoa per sample were assessed (per field of view). The following motility parameters were determined with the IVOS analyzer: total motility (TMOT, %), progressive motility (PMOT, %), average path velocity (VAP, μm/s), straight line velocity (VSL, μm/s), curvilinear velocity (VCL, μm/s), amplitude of lateral head displacement (ALH, μm), beat cross frequency (BCF, Hz), straightness (STR, ratio of VSL/VAP × 100%), and linearity (LIN, ratio of VSL/VCL × 100%). The analysis involved software settings recommended by the manufacturer for equine sperm: frame acquired—40, frame rate—60 Hz, minimum cell contrast—80, minimum cell size—3 pixels, straightness threshold—75%, path velocity threshold (VAP)—50 μm/s, low VAP cut-off—20 μm/s, low VSL cut-off—0 μm/s, static size gates—0.59–2.99, static intensity gates—0.68–1.74, and static elongation gates—12–97. The spermatozoa were considered progressively motile at VAP > 50.0 μm/s and STR > 75%.

#### 2.3.2. Spermatozoa with Normal Apical Ridge Acrosomes (NARs)

The percentage of spermatozoa with NARs was assessed using the Giemsa staining method described by Fraser et al. [24]. A minimum of 200 sperm cells per slide were examined under a bright-field microscope (Olympus CH 30, Olympus Corporation, Tokyo, Japan) at 1000× magnification and classified as spermatozoa with NARs (intact acrosomes) or as spermatozoa with damaged apical ridges. Spermatozoa with intact acrosomes had a uniform Giemsa staining pattern in the acrosomal region, whereas spermatozoa with damaged acrosomes had a patchy staining pattern with damaged apical ridges or loose acrosomal caps.

#### 2.3.3. Plasma Membrane Integrity (PMI)

Sperm plasma membrane integrity (PMI) was assessed using fluorochromes SYBR-14 and PI (Live/Dead Sperm Viability Kit; Molecular Probes, Eugene, OR, USA), according to a previously described method [25] with some modifications. Sample preparation and staining procedures have been described previously [26]. Stained sperm were examined under a fluorescence microscope (Olympus BX 41, Olympus Corporation, Tokyo, Japan) at 600× magnification. The microscope is equipped with filters with different excitation and emission wavelengths: UV (330–385 nm), blue (460–490 nm), and green (510–550 nm). SYBR-14 has an excitation peak at 488 nm and an emission peak at 518 nm when bound to DNA (green fluorescence). In turn, the PI fluorochrome has an excitation peak at 536 nm and an emission peak at 617 nm (red). At least 200 sperm cells were evaluated in each sample. The results are presented as a percentage of live sperm (sperm cells with green heads).

#### 2.3.4. Mitochondrial Membrane Potential (MMP)

Mitochondrial membrane potential (MMP) was assessed using fluorochrome JC-1 (Molecular Probes, Eugene, OR, USA), according to a previously described method [26] with some modifications. The working solution of JC-1 was prepared by dissolving 1 mg of JC-1 in 1 mL of anhydrous dimethyl sulfoxide. This fluorochrome is present at high concentrations in active mitochondria and forms aggregates displaying orange-red fluorescence (Ex/Em: 585/590 nm). In cells with low mitochondrial activity, JC-1 is present at low concentrations as a monomer and exhibits green fluorescence (Ex/Em: 510/527 nm). The applied sample preparation and staining procedures have been described previously [26]. Sperm samples were incubated at 37 °C for 15 min. After incubation, the PI solution (0.5 mg of PI/mL of phosphate-buffered solution, PBS) was added to the incubation mixture, and the samples were incubated for another 5 min at 37 °C. Stained cells were analyzed under a fluorescence microscope (Olympus BX 41, Olympus Corporation, Tokyo, Japan) at 600× magnification. At least 200 cells were evaluated in each sample. The results were expressed as the percentage of live sperm with high MMP (percentage of spermatozoa with a green head and an orange midpiece in the total population of stained sperm).

#### 2.3.5. Adenosine Triphosphate (ATP) Content

ATP content was determined with the ATP Bioluminescence Assay Kit CLSII (Roche Molecular Biochemical Company, Mannheim. Germany) and a Junior luminometer (Berthold Technologies, GmbH & Co., KG, Bad Wildbad, Germany). The procedure of extracting and quantifying ATP was described previously [27].

### 2.4. Statistical Analysis

The data were examined by repeated measures analysis of variance (ANOVA) using a general linear model procedure in Statistica v. 13.1 software (StatSoft Incorporation, Tulsa, OK, USA). The results were tested for normal distribution by the Shapiro–Wilk W-test. Levene’s test was used to assess the homogeneity of variances. The data were not normally distributed and were transformed accordingly. The values expressed as percentages (TMOT, PMOT, LIN, STR, NAR, PMI, and MMP) were subjected to arcsine transformation, and sperm motility parameters (VCL, VSL, VAP, ALH, and BCF) were log-transformed to obtain normally distributed data for one-way ANOVA. Differences between storage times and two seasons were analyzed using Tukey’s post hoc test. All results were expressed as the mean ± standard error of the mean (SEM) and were considered significant at *p* ≤ 0.05. The main effects of storage time and season, and their interaction effect on sperm quality characteristics, were analyzed using two-way mixed-model (4 × 2) ANOVA.

## 3. Results

### 3.1. Effect of Season on the Parameters of Fresh Stallion Ejaculates

The effect of season on the parameters of fresh stallion ejaculates (before dilution) is presented in Table 1. The ANOVA revealed that season significantly influenced ejaculate volume (Vol) and sperm concentration (Con). The ejaculates collected in spring/summer (breeding season) were characterized by larger volume, whereas the samples collected in fall/winter (non-breeding season) were characterized by higher sperm concentration and somewhat higher protein content. Sperm motility (MOT) was higher during the breeding season, but no significant differences (*p* ≥ 0.05) in this parameter were observed between the analyzed seasons.

### 3.2. Effect of Season and Storage Time on the Parameters of Cooled Stallion Spermatozoa

The values of progressive motility (PMOT), plasma membrane integrity (PMI), and mitochondrial membrane potential (MMP) were significantly higher (*p* ≤ 0.05) on the first (0 h) and second (24 h) day of storage during the breeding season than during the non-breeding season (Table 2). In addition, the percentage of spermatozoa with normal acrosomes (NARs) after 24 h of storage was higher during the breeding season than during the non-breeding season. No significant differences in TMOT and ATP values were observed between seasons regardless of storage time. Most of the examined parameters decreased on successive days of storage, and the noted decrease was also affected by season. During the breeding season, the values of NAR, PMI, and MMP decreased significantly after 24 h of storage, whereas during the non-breeding season, the above values decreased only after 48 h of storage. The values of TMOT and PMOT decreased significantly after 48 h of storage regardless of season.

**Figure 1:**
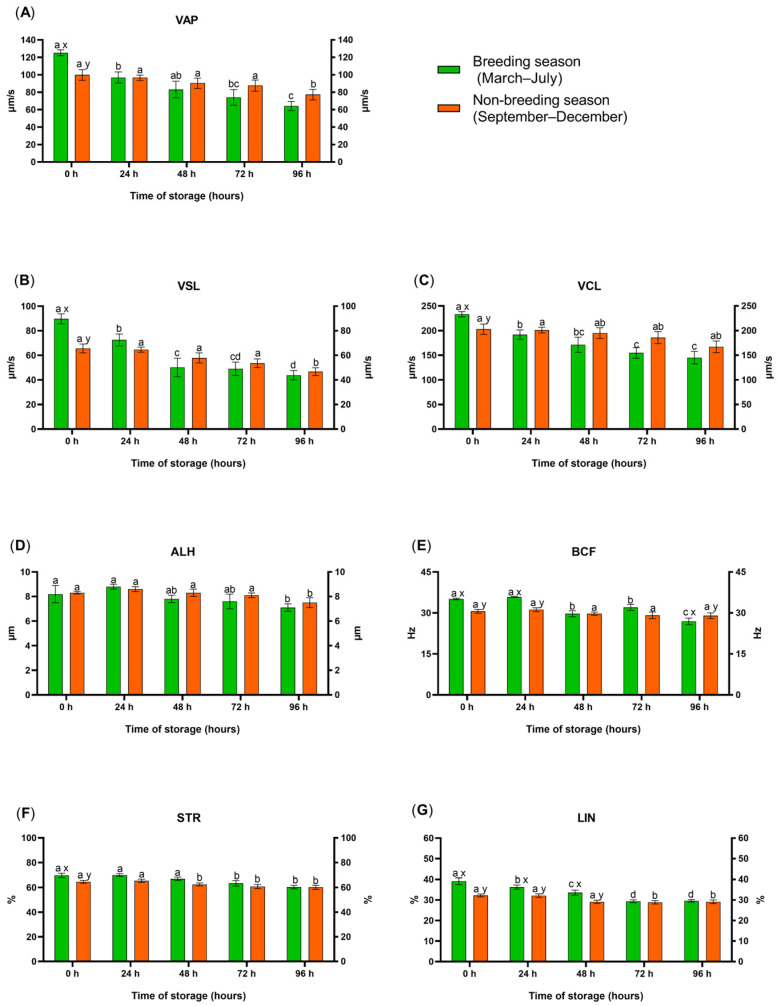
Effect of season on the kinematic parameters of motility: (**A**) VAP, average path velocity; (**B**) VSL, straight line velocity; (**C**) VCL, curvilinear velocity; (**D**) ALH, mean amplitude of lateral head displacement; (**E**) BCF, beat cross frequency; (**F**) STR, straightness, (**G**) LIN, linearity of stallion spermatozoa stored in the EquiPro extender at 5 °C for different periods of time. Values represent the means of (±SEM). Values marked with different letters (x, y) denote significant differences (*p* ≤ 0.05) between seasons. Values marked with different letters (a, b, c, d) denote significant differences (*p* ≤ 0.05) between storage times in the same season.

The analysis of the kinematic parameters of sperm motility revealed that average path velocity (VAP), straight line velocity (VSL), curvilinear velocity (VCL), beat cross frequency (BCF), straightness (STR), and linearity (LIN) values on the first day of storage (0 h) were higher during the breeding season than during the non-breeding season (Figure 1A,B,C,E,F,G, respectively). During the breeding season, the values of BCF and LIN were also higher after 24 h, and the values of LIN were also higher after 48 h of storage. In turn, after 96 h of storage, BCF values were significantly higher during the non-breeding season than during the breeding season. Most of the examined parameters decreased on successive days of storage, and the noted decline was also influenced by season. During the breeding season, a significant decrease in VAP, VSL, VCL, and LIN values was noted after 24 h of storage. In turn, during the non-breeding season, VAP, VSL, and VCL values decreased significantly after 96 h, and STR and LIN values decreased significantly after 72 h of storage. The values of BCF decreased significantly after 48 h of storage only during the breeding season. The percentage of spermatozoa moving in a straight line decreased after 48 h of storage during the non- breeding season and after 96 h of storage during the breeding season. A significant decrease in ALH values (Figure 1D) was noted after 96 h of storage regardless of season.

### 3.3. Two-Way ANOVA Analysis

The ANOVA revealed that season significantly affected PMOT, PMI, and MMP values, whereas storage time significantly influenced all of the studied variables (Table 3). In addition, PMI values were significantly (*p* ˂ 0.05) influenced by the interaction between storage time and season.

The analysis of the kinematic parameters of sperm motility (Table 4) revealed that VSL, BCF, and LIN were significantly influenced by season (*p* ˂ 0.001). Storage time significantly affected all of the examined motility parameters. In addition, VAP, VSL, VCL, and BCF were significantly influenced by the interaction between storage time and season (*p* ˂ 0.001). A significant interaction effect of storage time and season on LIN values was also observed (*p* = 0.041).

## 4. Discussion

Previous studies on the effect of season on the quality of stallion semen stored in a liquid state are limited, and those that are available have been conducted in other geographical locations. Furthermore, these studies primarily focused on the assessment of motility, membrane integrity, and DNA integrity [14,18]. The comprehensive analyses performed in this work (including motility, integrity of acrosomal and plasma membranes, mitochondrial activity, and the ATP content of spermatozoa) can expand the existing knowledge on the technological suitability of warmblood stallions’ sperm, collected during and outside the breeding season, for cold storage.

Season is one of the factors that must be considered in the process of optimizing AI techniques in horse breeding. The present study demonstrated that season significantly influenced the overall quality of warmblood stallion ejaculates. The ejaculates collected in spring/summer (March–July, breeding season) were characterized by larger volume, whereas those sampled in fall/winter (September–December, non-breeding season) were characterized by higher sperm concentration and somewhat higher total protein content. Similar observations were previously made by Shawki et al. [28], who found that ejaculate volume was significantly larger in spring and sperm concentration was higher (*p* < 0.05) in fall/winter, whereas total sperm counts did not differ significantly between seasons. Gamboa et al. [5] also reported higher sperm concentration and higher sperm progressive motility between September and December than between mid-May and July. In turn, Wach-Gygax et al. [18] and Janet et al. [15] observed the largest ejaculate volume and the lowest sperm concentration in summer. Contrary results have also been reported, but these studies were conducted on stallions in a different climatic zone [29,30]. Seasonal variations in ejaculate quality could be attributed to many factors, including individual traits [31], breed, and climate (including day length and temperature), which should be considered to ensure that ejaculates are used most effectively [15,16,18]. In this study, the percentage of motile spermatozoa did not differ significantly between the compared seasons, as previously shown by Janett et al. [15]. However, due to a considerable distance between the studs and the laboratory in this study, the analysis of sperm motility in raw semen was performed using the subjective method, which could have influenced the results.

Immediately after collection (and basic analyses), the ejaculates were diluted with extender to create the optimal conditions for storage. This procedure is carried out to protect stallion spermatozoa against seminal plasma components that can decrease the quality of sperm cells during prolonged storage [2,32,33]. The quality of fresh semen is a critical determinant of spermatozoa’s suitability for further storage.

The present study demonstrated that season and storage time significantly affected most quality parameters of spermatozoa stored in a liquid state for up to 96 h. A comparison of the ejaculates collected during the breeding and non-breeding seasons revealed the greatest differences in the quality of cooled spermatozoa on the first day of storage. The quality of extended semen on the first day of storage (0 h) was higher during the breeding season than during the non-breeding season, as reflected by the higher values in most quality parameters (PMOT, PMI, MMP) and motility parameters (VAP, VSL, VCL, BCF, STR, and LIN). These results corroborate the findings of Crespo et al. [31], who reported that season clearly influenced all analyzed parameters of semen collected from stallions of different breeds. Ejaculates sampled in the spring months were characterized by a higher percentage of progressively motile spermatozoa, a higher percentage of spermatozoa with integral plasma membranes, and a lower percentage of spermatozoa with fragmented DNA than the samples collected in the remaining seasons. According to Ebel et al. [34], sperm viability in the semen of heavy draft stallions was significantly higher during the breeding season compared with the non-breeding season. In turn, Jasko et al. [16] reported that season significantly influenced the motility parameters of stallion spermatozoa in a computer-aided sperm analysis. In the cited study, the percentage of motile and progressively motile spermatozoa, average path velocity, and mean straight line velocity were lower in winter than in spring/summer. Suliman et al. [35] observed significant seasonal variations in the percentage of normal sperm and the values of TMOT and VCL in ejaculates collected from fertile stallions during the breeding and non-breeding seasons (*p* < 0.05). They also found that VAP, VSL, and BCF values in the ejaculates collected from both fertile and infertile stallions were significantly higher during the breeding season.

In the current study, season exerted a significant effect on PMOT, NAR, PMI, and MMP values after 24 h of storage. This observation could suggest that season had the greatest impact on plasma membrane integrity, mitochondrial membrane potential, and consequently, sperm motility parameters. Changes in these structures can disrupt sperm motility, including a decrease in the motility parameters of spermatozoa stored in a liquid state at 5 °C [36,37]. According to other authors, the motility and viability of stallion spermatozoa could also decrease outside the breeding season due to DNA fragmentation and growing Ca^2+^ concentration in sperm cells [18,38]. The energy required for sperm motility is supplied by ATP molecules produced during glycolysis and oxidative phosphorylation processes in mitochondria. Oxidative phosphorylation is the dominant process in stallion spermatozoa [39]. Only spermatozoa that are progressively motile and have active mitochondria can successfully fertilize an egg cell [40]. Progressive motility is determined by VAP, VSL, and STR. In the present study, most motility parameters decreased gradually on successive days of storage, which is consistent with the findings of other researchers [18]. Interestingly, despite the fact that sperm motility decreased significantly during storage, no such changes were observed in ATP content, and even a minor increase in ATP levels was noted up to 72 h of storage. These results are difficult to interpret. It can be assumed that the generated ATP is not fully utilized by spermatozoa, and excess ATP remains in sperm cells. This observation could also suggest that in spermatozoa stored in a liquid state at a temperature of 5 °C, metabolic processes continue to occur up to 72 h of storage, as previously demonstrated in liquid-stored boar spermatozoa [27].

Regardless of storage time, the values of the examined parameters were generally higher during the breeding season than during the non-breeding season, which could be attributed to higher initial quality of stallion spermatozoa. However, the storage induced decrease in the values of the studied parameters (NAR, PMI, MMP, VAP, VSL, VCL, BCF, and LIN) was observed much earlier during the breeding season (after 24 h) than during the non-breeding season (after 48 h or later). Wach-Gygax et al. [18] observed improved sperm motility and the integrity of membranes of sperm in semen processed in the fall-winter months (January and October) and stored for 48 h compared to other months (July and February, respectively). In the work of Schmidt et al. [14], season did not affect the quality of spermatozoa in chilled and cryopreserved semen of Shetland pony stallions, although significant seasonal variations were observed in fresh spermatozoa. In turn, Blottner et al. [41] found that stallion ejaculates sampled during and outside the breeding season were characterized by similar sperm viability parameters after thawing, despite the fact that significant seasonal variations in sperm motility and morphology were determined before freezing. Therefore, the results of the current study could suggest that the spermatozoa of warmblood stallions collected during the non-breeding season are more resistant to cold shock compared with those collected during the breeding season. These observations suggest that spermatozoa sampled during and outside the breeding season and stored in a liquid state are characterized by similar suitability for reproductive purposes. However, to validate the present findings, the fertilizing capacity of stored sperm should be assessed in the future. The fertilizing capacity of spermatozoa can be determined by analyzing their ability to undergo capacitation and hyperactivation [42]. Both processes are essential for in vitro fertilization in horses. Further research is needed to address this issue.

## 5. Conclusions

Warmblood stallion spermatozoa sampled during the breeding season are characterized by higher initial quality than those collected outside the breeding season. However, an analysis of spermatozoa stored in a liquid state at a temperature of 5 °C demonstrated that sperm cells sampled during the non-breeding season (September to December) are more resistant to cold shock than those collected during the breeding season (March to July), which increases their suitability for cold storage. The study demonstrated that stallion spermatozoa collected in both seasons are characterized by similar suitability for liquid storage. The fertilizing capacity of stored spermatozoa should be examined in the future to confirm the present findings.

## Figures and Tables

**Table 1:** Effect of season on the parameters of stallion ejaculates.

Parameters	Breeding Season (March–July)	Non-Breeding Season (September–December)
Vol (mL)	74.7 ± 10.7 ^a^	66.1 ± 6.6 ^b^
Con (×10^6^/mL)	261.1 ± 37.5 ^a^	335.1 ± 87.2 ^b^
TP (mg/mL)	22.4 ± 4.7 ^a^	25.7 ± 6.3 ^a^
MOT (%)	75.0 ± 4.8 ^a^	70.8 ± 4.9 ^a^

Values marked with different letters (a, b) denote significant differences (*p* ≤ 0.05) between seasons. Vol—volume of ejaculate; Con—sperm concentration; TP—total protein content of seminal plasma; MOT—sperm motility.

**Table 2:** Effect of season on the total motility (TMOT), progressive motility (PMOT), acrosome integrity (NAR), plasma membrane integrity (PMI), mitochondrial membrane potential (MMP), and ATP content of stallion spermatozoa (mean ± S.E.M.) stored in the EquiPro extender at 5 °C for different periods of time.

Storage Time (h)	Breeding Season (March–July)	Non-Breeding Season (September–December)
TMOT (%)		
0	83.8 ± 2.9 ^a^	79.9 ± 2.2 ^a^
24	76.3 ± 5.4 ^ab^	74.6 ± 1.8 ^ab^
48	72.0 ± 5.9 ^b^	64.6 ± 3.5 ^b^
72	56.6 ± 6.3 ^bc^	55.3 ± 4.1 ^bc^
96	47.8 ± 4.2 ^c^	44.4 ± 4.4 ^c^
PMOT (%)		
0	43.1 ± 3.8 ^a x^	30.9 ± 2.8 ^a y^
24	39.4 ± 3.7 ^a x^	31.6 ± 2.6 ^a y^
48	30.1 ± 3.6 ^b^	24.4 ± 2.4 ^b^
72	19.0 ± 2.4 ^c^	17.5 ± 2.3 ^bc^
96	17.6 ± 2.3 ^c^	12.8 ± 1.8 ^c^
NAR (%)		
0	88.0 ± 0.9 ^a^	85.0 ± 2.0 ^a^
24	84.3 ± 1.6 ^b x^	79.5 ± 2.6 ^ab y^
48	82.5 ± 2.1 ^b^	79.4 ± 1.9 ^ab^
72	80.3 ± 1.9 ^bc^	75.7 ± 1.4 ^b^
96	70.0 ± 3.6 ^d^	70.3 ± 1.7 ^b^
PMI (%)		
0	89.6 ± 2.1 ^a x^	74.5 ± 1.2 ^a y^
24	79.1 ± 4.2 ^b x^	69.1 ± 2.3 ^ab y^
48	65.1 ± 2.2 ^c^	62.9 ± 2.7 ^b^
72	59.1 ± 4.1 ^bc^	58.4 ± 4.4 ^b^
96	53.2 ± 5.7 ^c^	48.3 ± 3.9 ^c^
MMP (%)		
0	87.5 ± 2.2 ^a x^	73.4 ± 1.8 ^a y^
24	76.4 ± 3.6 ^b y^	66.7 ± 2.2 ^a y^
48	63.1 ± 0.8 ^bc^	60.2 ± 2.4 ^b^
72	59.6 ± 3.8 ^c^	52.2 ± 4.4 ^bc^
96	55.9 ± 2.5 ^c^	50.1 ± 3.8 ^c^
ATP (nmol/10^8^ sperm)		
0	2.9 ± 0.8 ^ab^	3.5 ± 0.6 ^ab^
24	3.3 ± 0.6 ^ab^	3.8 ± 0.5 ^a^
48	4.2 ± 0.9 ^a^	3.3 ± 0.4 ^ab^
72	4.5 ± 0.6 ^a^	3.2 ± 0.5 ^ab^
96	2.4 ± 0.4 ^b^	2.4 ± 0.3 ^b^

Values in rows marked with different letters (x, y) denote significant differences (*p* ≤ 0.05) between seasons. Values in columns marked with different letters (a, b, c, d) denote significant differences (*p* ≤ 0.05) between storage times.

**Table 3:** Sources of variation in the quality parameters of stallion spermatozoa in extended semen, as determined in ANOVA.

Sources of Variations	d.f.	TMOT		PMOT		PMI		NAR		MMP		ATP	
		F-Value	*p*-Value	F-Value	*p*-Value	F-Value	*p*-Value	F-Value	*p*-Value	F-Value	*p*-Value	F-Value	*p*-Value
Time	4	10.671	0.001	16.802	0.001	7.894	0.001	2.648	0.035	13.084	0.001	7.600	0.001
Season	1	1.823	NS	7.080	0.008	9.133	0.002	0.120	NS	10.033	0.001	3.090	NS
Time × season	4	0.693	NS	2.280	NS	2.778	0.028	1.00	NS	1.621	NS	12.66	NS

The interaction effect of storage time (0 h, 48 h, 72 h, 96 h) and season (breeding season—March–July, non-breeding season—September–December) as the fixed factors were determined in repeated measures mixed-model ANOVA. Abbreviations: NS, not significant at *p* ≥ 0.05.

**Table 4:** Sources of variation in the kinematic parameters of motility of stallion spermatozoa in extended semen, as determined in ANOVA.

Sources of Variations	d.f.	VAP		VSL		VCL		ALH		BCF		STR		LIN	
		F-Value	*p*-Value	F-Value	*p*-Value	F-Value	*p*-Value	F-Value	*p*-Value	F-Value	*p*-Value	F-Value	*p*-Value	F-Value	*p*-Value
Time	4	10.671	0.001	16.802	0.001	7.894	0.001	2.648	0.035	13.084	0.001	7.600	0.001	6.955	0.001
Season	1	1.823	NS	7.080	0.008	0.068	NS	0.120	NS	10.033	0.001	3.090	NS	15.350	0.001
Time × season	4	5.998	0.001	13.999	0.001	4.483	0.001	1.483	NS	6.713	0.001	1.030	NS	4.229	0.041

The interaction effect of storage time (0 h, 48 h, 72 h, 96 h) and season (breeding season—March–July, non-breeding season—September–December) as the fixed factors were determined in repeated measures mixed-model ANOVA. Abbreviations: NS, not significant at *p* ≥ 0.05.

## Data Availability

The data presented in this study are available on request from the corresponding author.
